# A Complete Survey of Glycoalkaloids Using LC-FTICR-MS and IRMPD in a Commercial Variety and a Local Landrace of Eggplant (*Solanum melongena* L.) and their Anticholinesterase and Antioxidant Activities

**DOI:** 10.3390/toxins11040230

**Published:** 2019-04-19

**Authors:** Filomena Lelario, Susanna De Maria, Anna Rita Rivelli, Daniela Russo, Luigi Milella, Sabino Aurelio Bufo, Laura Scrano

**Affiliations:** 1 Department of Sciences, University of Basilicata, Via dell’Ateneo Lucano, 85100 Potenza (PZ), Italy; filomena.lelario@unibas.it (F.L.); sabino.bufo@unibas.it (S.A.B.); 2 School of Agricultural, Forest, Food and Environmental Sciences, University of Basilicata, Via dell’Ateneo Lucano, 85100 Potenza (PZ), Italy; demariasusanna@libero.it (S.D.M.); annarita.rivelli@unibas.it (A.R.R.); 3 Spinoff Accademico BioActiPlant, Via dell’Ateneo Lucano, 85100 Potenza (PZ), Italy; 4 Department of Geography, Environmental Management & Energy Studies, University of Johannesburg, Auckland Park Kingsway Campus, Johannesburg 2092, South Africa; 5 Department of European and Mediterranean Cultures, University of Basilicata, Via San Rocco, 75100 Matera, Italy; laura.scrano@unibas.it

**Keywords:** *Solanum melongena* L., malonylated form, glycoalkaloids, secondary metabolites, solasonine, solamargine, malonyl-solamargine, acetylcholinesterase, antioxidant

## Abstract

Eggplant contains glycoalkaloids (GAs), a class of nitrogen-containing secondary metabolites of great structural variety that may have both adverse and beneficial biological effects. In this study, we performed a complete survey of GAs and their malonylated form, in two genotypes of eggplants: A commercial cultivated type, Mirabella (Mir), with purple peel and bitter taste and a local landrace, named *Melanzana Bianca di Senise* (Sen), characterized by white peel with purple strip and a typical sweet aroma. Besides the analysis of their morphological traits, nineteen glycoalkaloids were tentatively identified in eggplant berry extracts based upon LC-ESI-FTICR-MS analysis using retention times, elution orders, high-resolution mass spectra, as well as high-resolution fragmentation by IRMPD. The relative signal intensities (i.e., ion counts) of the GAs identified in Mir and Sen pulp extracts showed as solamargine, and its isomers are the most abundant. In addition, anticholinesterase and antioxidant activities of the extracts were evaluated. Pulp tissue was found to be more active in inhibiting acetylcholinesterase enzyme than peel showing an inhibitory effect higher than 20% for Mir pulp. The identification of new malonylated GAs in eggplant is proposed.

## 1. Introduction 

*Solanum melongena* L., commonly known as eggplant or aubergine, is an economically important vegetable crop, belonging to the Solanaceae family, growing in tropical and temperate areas. It is the most widely consumed vegetable together with tomatoes and potatoes. The global production of eggplant has largely increased, reaching 52.3 million tons in 2017 [1]. This species includes a large number of commercial cultivars or varieties and local landraces that produce fruits differing in shape (ovoid, oblong, cylindrical, club-shaped), colour (purple, green, purple with white stripes) and size [2]. However, the elongated ovoid fruit with a dark purple/black peel is today the most-marketed worldwide, obtaining a general acceptance of its high nutritional value [3]. Eggplant is an inexpensive low-fat food source, providing energy, protein, fibre and vitamins, but it is actually studied as a source of health-promoting metabolites, including antioxidant and nutraceutical compounds, mainly anthocyanins and chlorogenic acid [4]. 

Moreover, eggplant also contains glycoalkaloids and saponins, which are responsible for the typical bitter taste of the pulp and are usually considered as anti-nutritional compounds, and are potentially toxic for humans as they can affect the absorption of nutrients [5]. Glycoalkaloids (GAs), a class of nitrogen-containing secondary metabolites, are commonly found in the Solanaceae family and play an important role in the defence of the plant against pests [6]. Although toxic for human health at certain levels, GAs also exhibit a wide range of pharmacological properties, including anticancer activity [7,8,9,10]. Many GAs exhibit acetylcholinesterase (AChE) inhibitory activity, which is associated with the treatment of several diseases such as Alzheimer’s disease (AD), *Myasthenia gravis*, and glaucoma as well as the mechanisms of insecticidal activity and anthelmintic drugs [11]. Furthermore, it has been demonstrated that the progression of neurodegenerative diseases is also related to the oxidative stress mechanism [12]. Several natural compounds have been shown to be useful tools for preventing oxidative stress and its damages and many plants have been studied, by different approaches, for the identification of new acetylcholinesterase inhibitors (AChE-Is). Both non-alkaloids and alkaloid-derivative compounds have been demonstrated to be new potential lead compounds for AD treatment [13].

**Figure 1:**
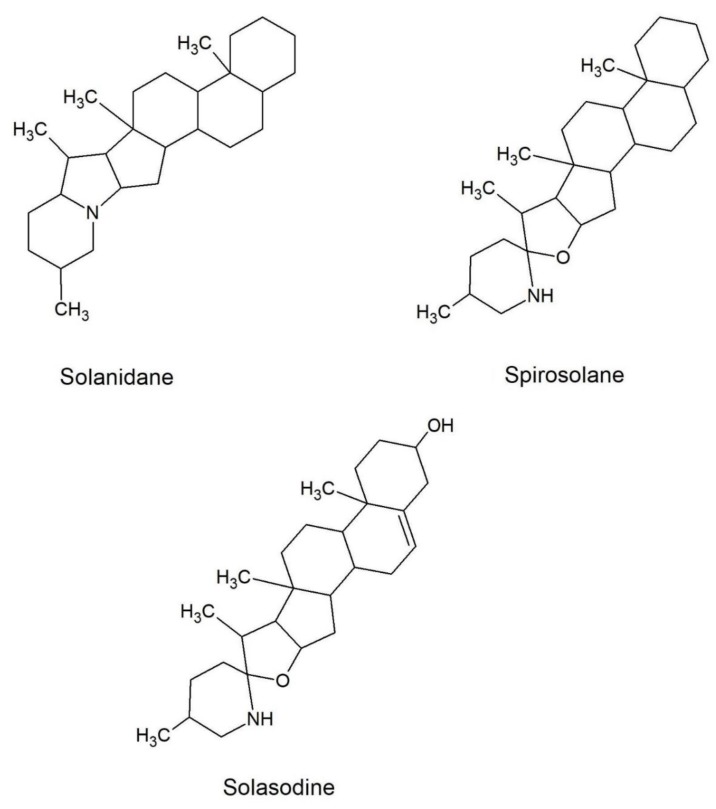
Basic structural formulas of the most common glycoalkaloid aglycons of *S. melongena*.

Thus, in recent years, medicinal uses of GAs have been the focus of scientific and pharmacological attention and their identification in plants has become a topic of increasing interest. However, nowadays most of the studies focus only on the presence of major GAs in each species, although it has been reported that accessions of the same species can have different GAs patterns (Figure 1) [14]. 

For common eggplant, only the two major GAs are usually reported and analysed: The spirosolane-type GAs solasonine and solamargine. Such GAs are structurally similar compounds and share the same aglycone, the solasodine, but differ in their carbohydrate component. They contain either glucose (solamargine) or galactose (solasonine) as the primary glycosylic residue. Both GAs, reported also in pepper, possess anti-proliferative activity on many human tumour cells [4]. 

Recently, some authors showed the occurrence of minor GAs and malonyl-GAs in Solanaceae plants. In particular, Wu et al. [15] showed the presence in *Solanum melongena* of minor GAs solanandaine, robeneoside B and 3’ or 6’ malonyl-solamargine by using reversed-phase LC-TOF-MS methods. The occurrence of a malonylated form of GAs in eggplant was also reported by Docimo et al. [16], which tentatively identified this compound as malonyl-solamargine based on the retention time data and by matching the MS/MS spectra with those reported in the *S. melongena* secondary metabolite database.

Nevertheless, a complete survey of glycoalkaloids and their malonylated form in the eggplant still needs to be recognized.

Several methods have been proposed for the identification and quantification of GAs in different species [17,18,19,20,21]. The presence of only small structural differences among various GAs requires the use of accurate and reproducible methods to identify and efficiently characterize them [22,23]. Electrospray ionization Fourier transform ion cyclotron resonance mass spectrometry (ESI-FTICR-MS) provides a highly selective tool for the unambiguous identification of molecules, which can be extended to minor components without significant interferences from other compounds in plant extracts [24,25,26]. In order to obtain a high degree of GAs structural information, infrared multiphoton dissociation (IRMPD) is widely used as the method of excellence, since it produces a larger number of fragments [27].

The hypothesis of this study was to verify the differences, by using a high-resolution LC-ESI-FTICR-MS method and IRMPD ion fragmentations, of the entire family of GAs and their malonylated forms in extracts from two types of eggplant grown in Mediterranean area beside a comparison of their Acetylcholinesterase (AChE) inhibitory and antioxidant activities. A commercial variety characterized by purple peel and bitter taste, and a local landrace, the *Melanzana Bianca di Senise*, recently included in the Traditional National Food Products (by the Italian Ministry of Agriculture Decree No. 168 issued on 17 June 2015), locally consumed and appreciated for the intense and fruity aroma of the berry, the sweetness and delayed turning brown of the pulp after cutting, have been used for this study.

## 2. Results and Discussion

### 2.1. Acetylcholinesterase Inhibition of Eggplant Extracts

The Solanaceae family contains members relevant to human nutrition and health. These include peppers, eggplant, tomato and potato as well as black nightshade and jimson weed seeds and tobacco. These plants produce different classes of compounds including alkaloids and glycoalkaloids (GAs). GAs exhibit also a wide range of pharmacological properties, including anticancer activity or acetylcholinesterase (AChE) inhibitory activity [7,8]. This latter is associated with the treatment of several diseases such as Alzheimer’s (AD) or Parkinson diseases. 

**Figure 2:**
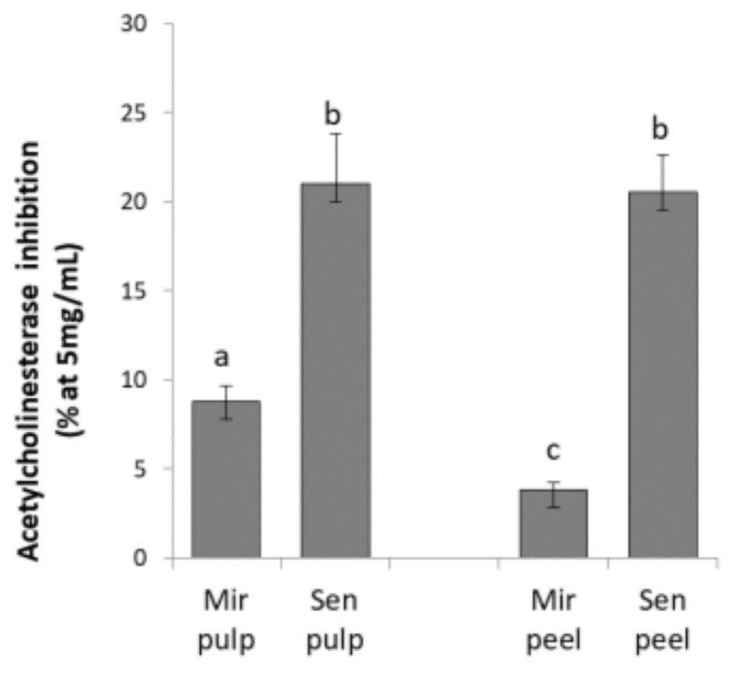
Inhibition of acetylcholinesterase (AChE) enzyme by pulp and peel aqueous extracts (1% acetic acid) from Senise (Sen) and common eggplant (Mir). Different letters indicate significant differences between mean values of a particular index of the given species *p* < 0.05 (according to Tukey’s test).

In this study, freeze-dried pulp and peel of two varieties of eggplant genotypes of *Solanum melongena* (Mirabella and Melanzana Bianca di Senise) were extracted by 1% (*v/v*) aqueous acetic acid solution. This extraction solution was used to recovery mainly glycoalkaloid compounds from plant tissues as reported by other studies [28]. Many species belonging to the Solanaceae family reported AChE inhibitory properties, but the importance of the chemical structure and the heterocyclic nitrogen of steroidal alkaloids play an important role in AChE inhibition [12]. Results of our study reported that peel and pulp extracts have a mild acetylcholinesterase inhibitory activity (Figure 2); no butyrylcholinesterase inhibition was shown at tested concentrations. The inhibition activity of the extracts was expressed as the % of inhibition at 5 mg/mL. Galanthamine was used as the reference standard and at the same concentration of extracts; it showed 100% AChE inhibition. Pulp tissue was found to be more active in inhibiting acetylcholinesterase enzyme than peel in Mirabella (Mir) sample (*p* < 0.05), so this part has been used for further analysis. 

### 2.2. GAs Profile of S. melongena var. Mirabella Pulp Extracts 

Due to the diversity of GAs in plants, which is considerably greater than previously thought, there is a demand to improve GAs identification methods. The direct analysis of secondary metabolites in plant extracts by reverse-phase liquid chromatography (LC) with electrospray ionization (ESI) and FTICR-MS has shown to be feasible in conjunction with IRMPD as a structural elucidation and/or confirmation tool [24,25,26,29].

The present work extends the previous efforts of investigators to elucidate the GAs profile of *Solanum melongena* L., which takes advantage of an optimized LC-ESI-FTICR-MS method. Separation and subsequent identification of GAs and their malonylated form were achieved upon direct extraction, using an aqueous acidified solution, and high-resolution mass spectral analysis of putative compounds [15,27]. Firstly, most naturally occurring GAs and malonyl-GAs of eggplant extracts were examined and characterized by MS and IRMPD MS^2^, then a comparison between the two genotypes was accomplished. 

Several minor GAs could be displayed by LC-ESI-FTICR-MS in positive ion mode through the narrow window extracted ion chromatograms (XICs) of each compound (± 1 mDa) from the complex matrix of berry pulp. This strategy decreased the background or co-eluted interferences in the chromatographic peaks. Surprisingly, XICs data analysis of peel extracts did not show the presence of a detectable level of GAs, so our study focused on pulp extracts. 

**Figure 3:**
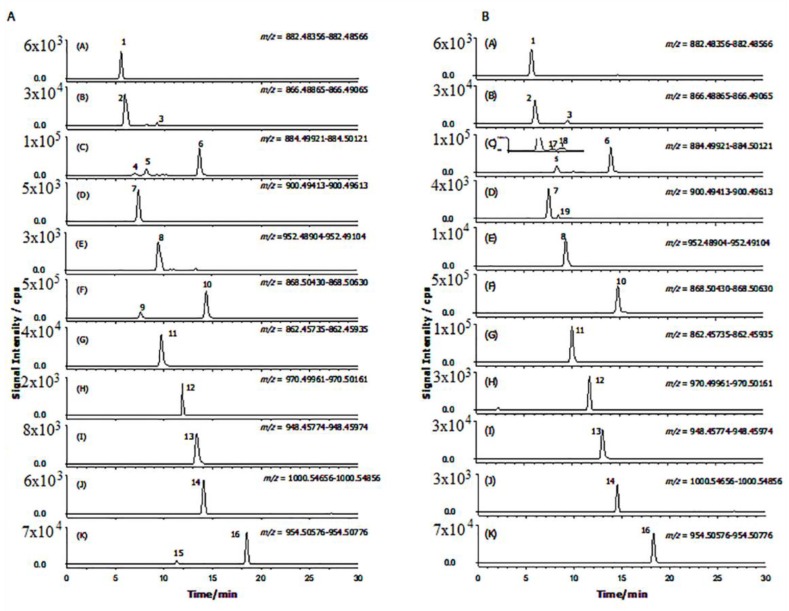
Extracted ion chromatograms (LC-ESI-FTICR-MS, high resolution) to the respective molecular ion peak [M+H]^+^ acquired in positive mode of “Mirabella” (**A**) and “Melanzana di Senise” (**B**) pulp extracts. The ion monitored are displayed in each trace (plots A–K) and peak numbers. Peak numbers are (1) solanidenetriol chacotriose, (2) solanidenediol chacotriose, (3) dehydrosolamargine, (4) solanandaine isomer I, (5) solanandaine, (6) solasonine (7) robenoside B, (8) malonyl-solanidenediol chacotriose, (9) solamargine isomer, (10) solamargine, (11) solanidatetraenol chacotriose, (12) malonyl-solanandaine, (13) malonyl-solanidatetraenol chacotriose, (14) arudonine, (15) malonyl-solamargine isomer, (16) malonyl-solamargine, (17) solanandaine isomer II, (18) solanandaine Isomer III, (19) robenoside B isomer.

A representative XICs obtained for a Mirabella pulp extract is reported in Figure 3A. As shown in Figure 3A, sixteen main peaks corresponding to eleven GAs and five malonyl-GAs have been identified with accurate monoisotopic values. 

In Table 1, the common name, molecular formula of protonated compounds, monoisotopic exact value as [M+H]^+^ ion and retention time of all detected GAs and malonylated GAs (listed with the same peak number used to identify each compound in the XICs) are reported; the examined *Solanum melongena* genotype is included as well. 

Interestingly, accurate mass data of GAs and malonylated-GAs were found as protonated molecules, with a mass error lower than 1.3 ppm, suggesting a very good mass accuracy.

Among all types of GAs and malonyl-GAs found in pulp extracts, only solasonine and solamargine were identified using commercial standard compounds. Identification of the other compounds was based on chromatographic behaviour, accurate mass measurements, IRMPD MS^2^ analyses and comparison with data from literature. 

As frequently encountered for most plant secondary metabolites, rich fragmentation patterns were produced during ionization of GAs and malonyl-GAs, which provide sufficient resolution for a priori structure elucidation. By analysing the chromatographic behaviour and the MS and MS^2^ spectra of compounds 8, 12, 13, 15 and 16 according to related literature [15,27,30], we can assume that these compounds are malonylated form of compounds 2, 5, 11, 9, 10, respectively. Compared with non-malonylated GAs, their chromatographic behaviour is characterized by higher retention time (Table 1).

On the basis of the above considerations, peak numbers were tentatively attributed to (**1**) solanidenetriol chacotriose, (2) solanidenediol chacotriose, (3) dehydrosolamargine, (4) solanandaine isomer I, (5) solanandaine, (6) solasonine, (7) robenoside B, (8) malonyl-solanidenediol chacotriose, (9) solamargine isomer, (10) solamargine, (11) solanidatetraenol chacotriose, (12) malonyl-solanandaine, (13) malonyl-solanidatetraenol chacotriose, (14) arudonine, (15) malonyl-solamargine isomer, (16) malonyl-solamargine, (17) solanandaine isomer II, (18) solanandaine isomer III. According to our knowledge, compounds 1, 2, 8, 11, 12, 13, 14 and 15 have not been previously described in eggplant.

It should be noted that there is a direct relationship between the level of malonyl-GAs and the level of correspondent-free GAs; thus, the relatively high intensity of solamargine also accounts for the high signal intensity of its malonyl conjugated compound. 

Noticeably, only the compounds that contain chacotriose as a carbohydrate component showed the formation of malonylated forms. This is probably due to their presence only in chacotriose of a glucose moiety as the directly joined to the aglycone (vide infra). We suppose that malonylation can occur to the primary OH-group of the glucose more easily than to the OH- located in other positions. Unsurprisingly, the peak signals of solasonine 6 and solamargine 10 were the most intense (Figure 3A). As previously demonstrated [27], ESI-IRMPD in positive ion mode of solasonine and solamargine led to the formation of numerous diagnostic signals (Supplementary Table S1). In particular, solasonine showed signals at accurate *m/z* values of 720.43324, 558.37856 and 396.32658 due to the sugar residue losses from dehydrated solasonine: The ion at accurate *m/z* 720.43324 corresponding to the loss of a rhamnose moiety (Rha, 146 Da), one of the three sugars composing the aglycone-linked solatriose, through glycosidic cleavage. The ion at *m/z* 558.37856 resulted from the loss of a glucose moiety (Glc, 162 Da) and the ion at *m/z* 396.32658 from the further loss of galactose (Gal, 162 Da).

Solamargine underwent similar fragmentation pathways as 6, generating characteristic fragments at accurate *m/z* values of 704.44910 ([M+H–H_2_O–Rha]^+^), 558.37844 ([M+H–H_2_O–2Rha]^+^) and 396.32617([M+H–H_2_O–2Rha–Glc]^+^) due to the sequential loss of the sugar residues composing chacotriose sugar chain. As expected, for both compounds 6 and 10, the ion at nominal *m/z* 414 (accurate *m/z* 414.33592 and 414.33688 for solasonine and solamargine, respectively) corresponding to protonated solasodine aglycone was detected.

The unknown peak with accurate *m/z* 866.48965 (3) exhibited a fragmentation pathway similar to that of solamargine. As reported in Supplementary Table S1, accurate mass measurements of the main diagnostic ions under laser irradiation dissociation of compound 3 led to molecular formula fragments with two hydrogen atoms less than those detected for solamargine (10). Therefore, this GA is probably a dehydrogenated form of solamargine, where a chacotriose sugar chain is linked to an aglycone of nominal *m/z* 412 (accurate *m/z* 412.32129), which is likely a solasodine derivative with an additional double bond (molecular formula C_27_H_41_NO_2_). This hypothesis was confirmed by the elution order (Table 1) of compound 3 (Rt = 9.2 min), which eluted earlier than compound 10 (Rt = 14.3 min). In fact, it is known that an increase of double bonds in a given GA causes it to elute earlier. Based on the reported considerations, compound 3 is tentatively determined as dehydrosolamargine (C_45_H_72_NO_15_^+^, Δm = −0.2 ppm). 

An isomer of compound **3** eluting at Rt of 6.0 min was also displayed in the XICs trace of the protonated precursor ion at *m/z* 866.48965 (compound 2, C_45_H_72_NO_15_, Δm = 0.5 ppm). 

The characteristic IRMPD MS^2^ product ions reported for this compound (2) in Supplementary Table S1 at *m/z* 720.43188 ([M+H–Rha]^+^), 574.37402 ([M+H–2Rha]^+^) and 412.31111 ([M+H–2Rha–Glc]^+^) suggest the presence of the chacotriose moiety linked to the skeleton of solanidenediol aglycone (C_27_H_42_NO^+^, Δm = 0.2 ppm). Generally, solanidane GAs elute earlier than spirosolanes (Figure 1) and addition of hydroxy groups makes their structure even more polar, thus, compound 2, which should have two hydroxy groups on the aglycone, was characterized as solanidadienol chacotriose (Table 1). 

Similarly, the IRMPD MS^2^ mass spectrum of compound 1 at *m/z* 882.48419 shows the neutral losses of Rha and Glc sugars from chacotriose moiety yielding the aglycone ion at *m/z* 428.31628 (C_27_H_42_NO_3_). Subsequently, the aglycone undergoes sequential loss of three water molecule giving rise to the *m/z* 374.28439 ion. This, together with the product ion at *m/z* 267.17441 (C_19_H_23_O^+^, Δm = 0.2), infers that in compound 1 an additional oxygen atom is localized to solanidadienol aglycone. Therefore, compound 1, which elutes before compound 2, was identified as a solanidenetriol chacotriose. 

Through the XICs of the protonated precursor ion at *m/z* 862.45835 in the full scan (±1 mDa), peak 11 was obviously observed at Rt of 9.7 min, providing an intensive [M+H]^+^ ion at *m/z* 862.45864 (C_45_H_68_NO_15,_ Δm = 0.3 ppm). The fragmentation (Supplementary Table S1) was consistent with that of a structure composed of a chacotriose sugar chain and an aglycone of nominal *m/z* 408 (C_27_H_38_NO_2,_ Δm= 0.1 ppm) which have not been previously reported in eggplant. The occurrence of *m/z* 716.39879 [M+H–Rha]^+^, *m/z* 570.34136 [M+H–2Rha]^+^ and *m/z* 408.28930 [M+H–2Rha–Glc]^+^ product ions correspond to the typical sequential loss of Rha-Rha-Glc already displayed for solamargine. 

One possibility is that this compound corresponds to solanidatetraenol chacotriose, which would account for a *m/z* 408.28930 (C_27_H_38_NO_2,_ Δm = −1.0 ppm) aglycone. To confirm this hypothesis, the elution time of compound 11 was compared to that of solamargine and the elution order was consistent with what would be the expected for a GA with additional double bonds [20]. 

Similar to the peak 11, the XICs of the protonated precursor ion at *m/z* 900.49513 in the MS full scan (±1 mDa) for *Mirabella* pulp extract showed the presence of a single chromatographic peak (7) at a Rt of 7.3 min (Figure 3A).

In Supplementary Figure S1, the IRMPD-FTICR mass spectrum of the precursor ion from peak 7 ([C_45_H_73_NO_17_]^+^, Δm = +0.6 ppm) is reported. It exhibits a remarkable fragmentation upon 290 ms of laser irradiation at the highest energy, leading to the formation of many diagnostic fragments. This compound eluting at 7.8 min and consisting of solatriose and an aglycone at nominal *m*/*z* 430 seems to be a hydroxyl-solasonine.

A typical set of signals already described for solasonine and due to the sequential neutral losses of Rha, Glc and Gal was detected. Product ions of *m*/*z* 430.33142 [M+H−solatriose]^+^, 412.32111 [M+H–H_2_O−solatriose]^+^, 394.31055 [M+H–2H_2_O−solatriose]^+^ and 376.26370 [M+H–3H_2_O−solatriose]^+^, corresponded to the sequential loss of the hydroxy groups. The order of elution of compound 5 in respect to solasonine (6) is also consistent with those expected for a GAs with an additional hydroxy group. 

Based on the suggested fragmentation pattern, literature data [15] supported by the derived elemental compositions from the accurate mass measurements of all the product ions (Supplementary Table S1) and taking into account that two GAs, namely, robeneoside A and robeneoside B have been isolated and identified from the fruits of *Solanum lycocarpum* [31], peak 5 was tentatively identified as robeneoside B.

Another compound with *m/z* 900.49628 was detected at 8.6 min only in the genotype *Melanzana Bianca di Senise* (Compound 19, Table 1). It underwent similar fragmentation pathways as 5, so it was tentatively identified as a 12-hydroxysolasonine. These results are in agreement with those reported in *Solanum lycocarpum* [31].

When subjected to IRMPD MS^2^ analysis, the protonated molecules of all malonyl-GAs share a characteristic set of neutral losses (Supplementary Table S1); these correspond to the loss of H_2_O (18.0106 Da) from the aglycone and chacotriose, CO_2_ (43.9892 Da) and C_2_H_2_O (42.01001 Da) from the malonyl group, one or two rhamnose (146.0579 Da = C_6_H_10_O_4_) and glucose (162.0528 Da = C_6_H_10_O_5_) from chacotriose. The neutral loss of the whole group C_3_H_2_O_3_ (85.99984 Da) was also observed in their MS^2^ mass spectra, which confirmed the presence of malonic acid (Supplementary Table S1). Thus, the spectra of malonylated-GAs were similar to those previously reported for the protonated molecules of the corresponding non-malonylated forms [27]. 

**Figure 4:**
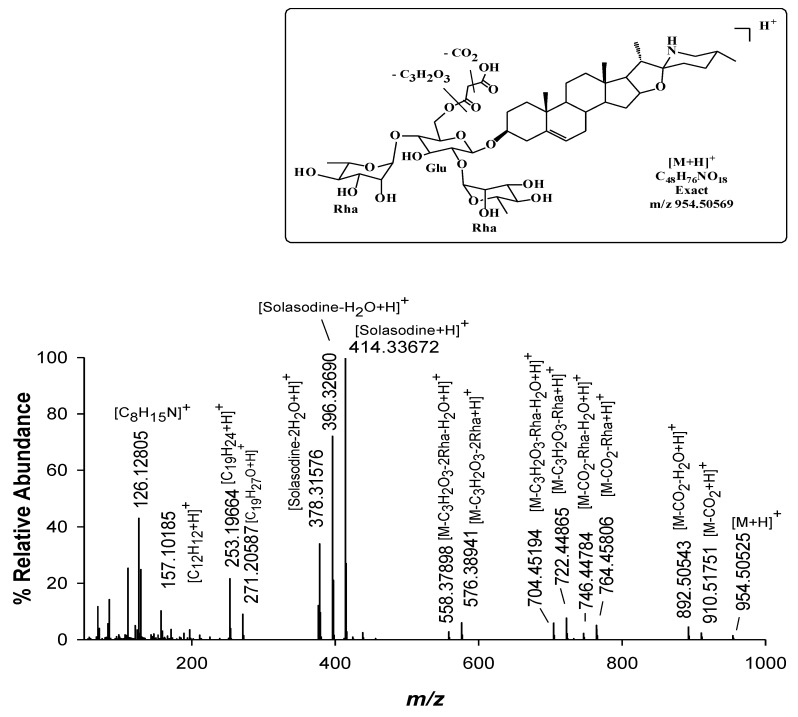
High-resolution IRMPD FTICR mass spectrum of the protonated malonyl-solamargine at *m/z* 954. Following transfer to the ICR cell, precursor ion populations were photon-irradiated for 290 ms at 100% laser power.

For example, the IRMPD MS^2^ spectrum of compound 16 (molecular formula of protonated molecule C_45_H_73_O_16_N, calculated for C_45_H_74_O_16_N, *m/z* 954.50569; Δm = 0.5 ppm), is similar to mass spectra of solamargine [27]. As summarized in Figure 4, in addition to the characteristic fragments already described for solamargine at *m/z* 576.38941 [M+H–2Rha]^+^, 558.37898 [M+H–2Rha–H_2_O]^+^, 414.33672 [M+H–2Rha–Glc]^+^, 396.32690 [M+H–2Rha–Glc–H_2_O]^+^, 378.31576 M+H–2Rha–Glc–2H_2_O]^+^, [C_19_H_27_O+H]^+^ 271.20587 and 253.19502 [C_19_H_25_+H]^+^, due to the fragmentation of solasodine aglycone and chacotriose sugar residue, the isolated protonated compound [M+H]^+^ at *m/z* 954.50525 under laser irradiation led to the formation of other diagnostic fragment ions. The occurrence of [M+H–CO_2_]^+^ at *m/z* 910.51751, [M+H–CO_2_–H_2_O]^+^ at *m/z* 892.50543, [M+H–CO_2_–Rha]^+^ at *m/z* 764.45806, [M+H–CO_2_–H_2_O–Rha]^+^ at *m/z* 746.44784 and [M+H–C_3_H_2_O_3_–Rha]^+^ at *m/z* 722.44865, suggests the presence of a malonate ester in the sugar group (Figure 4), most probably in glucose residue in position 3’ or 6’ [15]. This compound with molecular formula C_48_H_75_O_18_N and retention time of 18.5 min was tentatively assigned the identity of malonyl-solamargine. 

The location of the malonyl group in the sugar is not clear, but no neutral loss of terminal rhamnose with malonic ester was detected under fragmentation of malonyl-solamargine (Figure 4), consistent with the possibility that the malonyl groups are connected to glucose moieties in position 3’ or 6’ [15]. Since the occurrence of a malonyl ester in the glucosyl moiety causes a negative charge and could facilitate the efficient transport of these compounds into the vacuole by anionic transporters, malonylation of secondary metabolites is considered an important step in plant defence process [31]. 

Taking into account that in many secondary metabolites produced in Solanaceae plants and involved in plant defences against insect herbivory, malonyl moiety is typically connected to the C’-6 hydroxyl groups of the glucose [32,33,34,35] we can suppose that this is the preferential position of attachment of malonyl group also for GAs.

As shown in Figure 3A and Table 1, four structural isomers of solasonine (compounds 4, 5, 17 and 18) and one isomer of solamargine (compound 9, named solamargine isomer) have been displayed through the XICs of *m/z* 884.50021 (±1 mDa) and *m/z* 868.50530 (±1 mDa) in full-scan MS data obtained for the commercial variety *Mirabella* berry pulp extract analysis.

Compounds 4 and 9 were detected only in *Mirabella* while all the other compounds occurred in both *Mirabella* and *Melanzana Bianca di Senise* extracts.

Supplementary Figure S2 shows the IRMPD spectra of the charged precursor ions [M+H]^+^ at *m/z* 884.49982 (A) and [M+H]^+^ at *m/z* 970.50159 (B), tentatively assigned to solanandaine (5) and malonyl-solanandaine. The positive IRMPD ion spectrum of solanandaine showed a peak at accurate *m/z* 884.49982 [M+H]^+^ corresponding to the molecular formula C_45_H_73_O_16_N (calculated for C_45_H_74_O_16_N, *m/z* 884.50021; Δ*m* = −0.4 ppm). In addition, significant peaks at *m/z* 720.43724 ([M+H–H_2_O–Rha]^+^), 574.37463 ([M+H–H_2_O–2Rha]^+^), 430.33215 ([M+H–2Rha–Glc]^+^), 412.32132 ([M+H–H_2_O–2Rha–Glc]^+^) and 142.12265 ([C_8_H_15_NO+H]^+^) were detected. This GA, therefore, contains three hexose units. The sequential loss of Rha, Rha and Glc indicates that solanandaine has a chacotriose side chain attached to an aglycone moiety.

The aglycone *m/z* 430.33215 [Agly+H]^+^ is likely a solaparnaine, a solasodine derivative with an additional hydroxyl group in ring F, as confirmed by the occurrence of fragment at *m/z* 142.12265 ([C_8_H_15_NO+H]^+^) corresponding to the characteristic spirosolane fragment originated from ring E breaking at *m/z* 126.12769 ([C_8_H_15_N+H]^+^) plus an OH group. 

As expected, compound 12 showed the same fragmentation behaviour of solanandaine (5) due to the facile breaking of ester link, by the losses of CO_2_ (44 Da) and of an additional 42 Da-moiety (C_2_H_2_O) (Supplementary Figure S3). Additional neutral loss of 146 Da, 146 Da and 162 Da and accurate mass measurements of the remaining aglycone protonated at *m*/*z* 430.33176 (calculated *m*/*z* for C_27_H_44_NO_3_^+^ = 430.33157; Δm = 0.5 ppm) suggest the presence of a malonyl-chacotriose linked to solaparnaine aglycone.

The same rationale used for malonyl-solamargine and malonyl-solanandaine was applied to explain the occurrence of malonyl-solanidenediol chacotriose (8), malonyl-solanandaine (12), malonyl-solanidatetraenol chacotriose (13) and malonyl-solamargine isomer (15), presumably formed because of the relatively high content of solanidenediol chacotriose, solanandaine, solanidatetraenol chacotriose and solamargine isomer, peaks 2, 5, 11 and 9, respectively. To the author’s knowledge, the only detailed data on malonylated GAs compounds reported in eggplant, thus far, in the literature are those on malonyl-solamargine [15]. 

The more informative fragments in the IRMPD MS^2^ spectrum from the protonated ion [M+H]^+^ of compound 4 (which was of low intensity) were [M+H–H_2_O]^+^ at *m*/*z* 866.49023 and [M+H–H_2_O–chacotriose]^+^ at *m*/*z* 412.32104 in addition to the characteristic fragments of spirosolane type aglycone. The peak at nominal *m*/*z* 430 corresponding to aglycone solasodine, or its isomer, with an additional hydroxyl group, was not observed in the spectrum 4 but can be attributed to facile water loss. Therefore, we can suppose that compound 4 was an isomer of solanandaine. 

The same considerations could be done for compounds 17 and 18, named solanandaine isomer II and solanandaine isomer III, because their fragmentations (Supplementary Table S1) were consistent with a structure composed of chacotriose linked to an hydroxysolasodine or its isomer detected at *m/z* 430.33118 for compound 17 and at *m/z* 430.33173 for compound 18. 

Compound 14 was eluted at Rt = 14.1 min and the ESI-MS spectrum showed an [M+H]^+^ ion at accurate *m*/*z* 1000.54779 with an elemental composition of non-protonated compound C_50_H_81_NO_19_.

Although the mass spectrum was less rich in diagnostic fragments than other GAs shown above, the presence of a solasodine core linked to a tetrasaccharide moiety could be established.

The IRMPD MS^2^ mass spectrum (Supplementary Figure S2) gave fragments at *m*/*z* 982.53912 ([M+H–18]^+^) and *m*/*z* 836.47998 ([M+H–18–146]^+^), which correspond to the loss of a molecule of water and an additional rhamnose. The peaks at *m*/*z* 704.43732 ([M+H–18–132–146]^+^) and *m*/*z* 558.37921 ([M+H–18–132–146–146]^+^) corresponded to the loss from [M+H–18]^+^ of a xylose and a rhamnose, and a xylose and two rhamnose moieties, respectively. The fragment at *m*/*z* 414.33667 corresponding to the protonated aglycone ([solasodine + H]^+^) could be originated from the loss of the entire sugar moiety composed of a xylose, two rhamnose and a glucose.

Based on the mass difference between this compound and solasonine (132 Da), IRMPD data and according to a previous report [36], compound 14 was deduced as arudonine, an allelopathic steroidal glycoalkaloid found in the root bark of *Solanum arundo* Mattei.

Interestingly, no malonylated form of this GAs was detected, probably due to the low level of arudonine in the sample. 

### 2.3. GAs Profile of Melanzana Bianca di Senise Pulp Extracts 

In view of the worldwide interest in local germplasm conservation and considering the risks resulting from genetic erosion of agricultural plant resources, several research projects have addressed the safeguarding and conservation of agro-biodiversity [37,38], including endemic or local eggplant genotypes [39].

Local landraces, unlike the commercial varieties, are often poorly studied, and little scientific information is available on their characteristics and distinctive features, despite being generally associated with better flavour, local tradition and environmentally friendly production [40]. This is the case of *Melanzana Bianca di Senise*, an eggplant locally appreciated for the intense and fruity aroma of the berry, its sweetness and particularly for delaying of turning brown of the pulp after cutting, of which there are no studies in the literature.

For this reason, the optimized, positive-ion LC-FTICR-MS method used to characterize the commercial variety *Mirabella* was then used to profile and compare GAs from *Melanzana Bianca di Senise* pulp extracts. The resultant XICs are presented in Figure 3B. Identification of individual compounds, provided in Table 1 and Supplementary Table S1, revealed a profile very similar to that of Mirabella variety, lacking only compounds 9 and its malonylated form 15 and showing, as mentioned above, the occurrence of three additional compound in the XICs trace, one at *m/z* 900.49513 (±1 mDa), tentatively assigned to a robenoside B isomer, probably compound 12-hydroxysolasonine [31] and two at *m/z* 884.50021 (±1 mDa), tentatively assigned to Solanandaine Isomer II (spirosolendiol chacotriose) and Solanandaine Isomer III (spirosolendiol chacotriose).

On the basis of the GAs profile of the local eggplant landrace that resulted similar to Mirabella and considering the excellent recognized organoleptic characteristics (sweetness and delaying of turning brown of the pulp after cutting), the nutritional value of the *Melanzana Bianca di Senise* should be further analysed aiming at the protection and preservation of agro-biodiversity. 

### 2.4. Composition Profile of GAs in Solanum Melongena

As far as quantification of GAs in sample extracts of *Solanum melongena*, the use of solasonine and solamargine as calibration standards established that the LC-FTICR-MS method was linear over at least two orders of magnitude, 0.5–50 ppm (data not shown). Due to the lack of commercially available standards, only these two compounds were quantified. Solamargine was found the most representative with relatively high content, i.e., 37.3 ± 1.8 mg/100g (dry weight (dw), three samples); the level of solasonine was considerably lower than those of solamargine, i.e., 6.2 ± 0.5 mg/100g (dry weight (dw), three samples).

**Figure 5:**
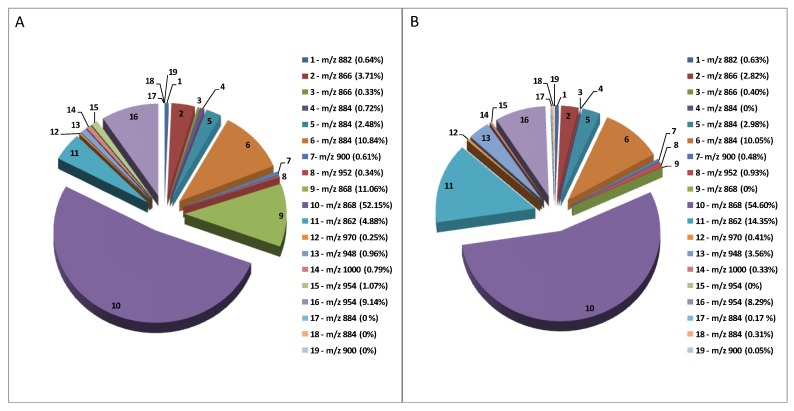
Pie charts illustrating the relative signal intensities (i.e. ion counts) of the GAs identified in “Mirabella” (**A**) and “Melanzana di Senise” (**B**) pulp extracts. (1) solanidenetriol chacotriose, (2) solanidenediol chacotriose, (3) dehydrosolamargine, (4) solanandaine isomer I, (5) solanandaine, (6) solasonine (7) robenoside B, (8) malonyl-solanidenediol chacotriose, (9) solamargine isomer, (10) solamargine, (11) solanidatetraenol chacotriose, (12) malonyl-solanandaine, (13) malonyl-solanidatetraenol chacotriose, (14) arudonine, (15) malonyl-solamargine isomer, (16) malonyl-solamargine, (17) solanandaine isomer II, (18) solanandaine Isomer III, (19) robenoside B isomer.

Figure 5 depicts the relative proportions in terms of ion counts of each GAs occurring in the Mirabella and Melanzana Bianca di Senise extracts. To the best of our knowledge, this is the broadest spectrum of GAs known to be identified in *Solanum melongena*. As expected, solamargine (peak 10, nominal m/z 868), solasonine (peak 6, nominal m/z 884) and malonyl-solamargine (peak 11, nominal m/z 954) are the most representative GAs in both pulp extracts, amounting to >70% of the total GAs present in samples. Interestingly, only in Mirabella extract it was possible to detect high level of a solamargine isomer (peak 9, nominal m/z 868), i.e 11.06%, and of the its manolynated form at nominal 954 (peak 15), i.e 1.07%; on the contrary, in the Melanzana Bianca di Senise it was possible to highlight the occurrence of a high level of solanidatetraenol chacotriose i.e 14.35%, (peak 11, nominal m/z 862) and its malonylated form (peak 13, nominal m/z 948), which are at low levels in the Mirabella variety. 

As presented, the method is basically semi-quantitative. However, by the use of appropriate calibration standards, it would be straightforward to convert the method into a fully quantitative procedure and, thus, provide precise concentrations of individual GAs in pulp extracts of *Solanum melongena*. 

### 2.5. Acetylcholinesterase Inhibition Activity of Eggplant GAs

Solamargine was tested to evaluate the cholinesterase inhibition (Supplementary Figure S4) and then compared to galanthamine to estimate its contribution to extract activity. Solamargine showed an IC_25_ of 327.88 µM = 0.28 mg/mL (IC_50_ was not reached at the tested concentrations), whereas galanthamine showed a higher value of inhibition (IC_25_ of 5.31 µM). A previous study reported that the pure steroidal glycoalkaloids α-solanine and α-chaconine significantly inhibited acetylcholinesterase enzyme, whereas solasonine and solamargine showed a very reduced activity [41]. Nevertheless, it was found that combinations of solanine and chaconine, and also of solasonine and solamargine, produced effects which were slightly antagonistic or non-interactive. This could explain why the extracts showed lower AChE inhibition than pure compounds. Referring to literature results, other GAs could be more active than solamargine in eggplant extracts. Our further studies will be directed to the isolation, and activity determination of other GAs identified in the extracts.

### 2.6. Antioxidant Activity of Eggplant Extracts

The antioxidant activity was also evaluated by different assays able to measure the radical scavenging activity of extracts and the possible inhibition of lipid peroxidation. The antioxidant activity was determined by widely used antioxidant methods as 2,2-diphenyl-1-picrylhydrazyl radical (DPPH) and beta carotene bleaching assay (BCB).

**Figure 6:**
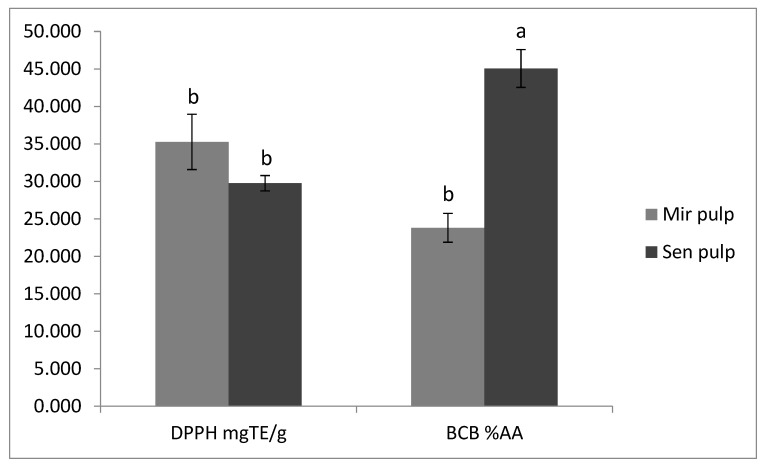
Antioxidant activity of the pulp extract from “Mirabella” and “Melanzana di Senise” samples. DPPH (2,2-diphenyl-1-picrylhydrazyl) results were expressed as mg of Trolox^®^ equivalent (TE) per mg of dried extract; BCB (beta Carotene Bleaching) results were expressed as % antioxidant activity (% A.A.). Different letters indicate significant differences between mean values of a particular index of the given species *p* < 0.05 (according to Tukey’s test).

The results of the antioxidant activity reported in Figure 6 did not demonstrate significant differences in DPPH assay (*p* > 0.05), while Melanzana di Senise was shown to be two folds more active than the Mirabella sample when the antioxidant activity was measured using the BCB test (45% *vs.* 23% respectively; *p* < 0.05). It was demonstrated that the antioxidant activity of Solanaceae species is not directly correlated to the amount of glycoalkaloids but, rather, to phenolic compounds [6]. This means that the antioxidant activity showed by the extract was preferably due to the presence of phenolic compounds solubilised in the extraction solvent (acidified water) together with GAs. 

## 3. Conclusions 

In conclusion, the investigation and the structural characterization of the GAs and malonyl-GAs in the two considered eggplant berries were performed successfully by LC-ESI-FTICR-MS. Assisted by the high mass accuracy, high-resolution and flexible MS/MS capability of a Fourier transform ion cyclotron resonance (FTICR) mass spectrometer, infrared multiphoton dissociation (IRMPD) fragmentation of GAs has been accomplished allowing the tentative identification of all compounds under investigation and showing the presence of some GAs and malonyl-GAs not previously reported for this vegetable. The study based on MS methods is a valuable source of information for further isolation and structural investigations (e.g., by NMR) aimed at elucidating new GA structures. Secondary metabolites identified in the plant extracts demonstrated to inhibit a key enzyme involved in several neurological disorders. Even that some GAs were demonstrated to be toxic in human and animal models, in other experiments a wide range of biological activities have been evidenced [7,8,9,10,42,43]. Our results reinforced the hypothesis that GAs could serve as lead compounds for the design of new drugs active vs. cholinesterase effective in neurological disorders. 

## 4. Materials and Methods 

### 4.1. Chemicals

Acetonitrile (ACN; LC-MS grade) and HCOOH (99%) were from Carlo Erba (Milan, Italy). Ultrapure H_2_O was produced using a Milli-Q system (Millipore, Billerica, MA, USA). Pure solamargine (97.5%) and solasonine (98.3%) were purchased from Glycomix (Reading, UK). 

Acetylthiocholine iodide, 5,5′-Dithiobis(2-nitrobenzoic acid) (DNTB), Tris-HCl, bovine serum albumin (BSA), acetylcholinesterase (AChE), butyrylcholinesterase (BChE), butyrylthiocholine chloride, galanthamine (99.0% purity), sodium carbonate, 2,2-diphenyl-1-picrylhydrazyl radical (DPPH), beta-carotene, linoleic acid, Trolox^®^ and Tween 20 were purchased by Sigma-Aldrich (Milan, Italy).

### 4.2. Plant Material and Sample Preparation 

The experiment was carried out on the berries of two eggplant genotypes of *Solanum melongena* L.: The commercial variety *Mirabella* (Mir) and the local landrace *Melanzana Bianca di Senise* (Sen), grown in an experimental field located in Rotonda (PZ, Italy) at the Experimental Agricultural Farm Station sited in the Pollino National Park. The plants were grown and samples were collected under special permission from the Farm Director to conduct the study on this area. This study did not involve endangered or protected species. For both genotypes, at the commercial fruit ripening stage, ten fruits from ten plants were harvested and cleaned, then morphological traits were recorded (peel colour, length, diameter and weight of the berries are shown in Supplementary Table S2). Subsequently, the fruits were longitudinally divided into four equal parts, peeled to separate the peel and pulp, quickly frozen in liquid N_2_, and lyophilized. The freeze-dried tissue samples were ground to a fine powder and stored at −80 °C until analysis for glycoalkaloids. The extraction procedure was based on the method of Cataldi, Lelario and Bufo [28]: A 1.5 g powdered sample was extracted with 20 mL of 1% (*v/v*) aqueous acetic acid solution. The suspension was mixed for about two hours to facilitate the passage of soluble substances in the extraction solvent and subsequently centrifuged at 6000 rpm for 30 min. The supernatant was collected using a syringe and filtered through a 0.22 μm nylon filter (Whatman, Maidstone, UK). Afterwards, the pellet was re-suspended in 5 mL of 1% (*v/v*) aqueous acetic acid solution, stirred, centrifuged, collected and then filtered. The two extracts were joined in a unique sample.

### 4.3. Analysis of Glycoalkaloids (GAs)

GAs analyses were performed using a Surveyor autosampling LC system, interfaced to a LIT-FTICR-mass spectrometer (Thermo Fisher Scientific, Bremen, Germany) via electrospray source, equipped with a 20 W CO_2_-laser (Synrad, Mukilteo, WA, USA; 10.6 μm) described elsewhere [27]. Source settings used for the ionization of GAs were ESI needle voltage, +4.5 kV; capillary voltage, +35 V; temperature of the heated capillary, 300 °C; and sheath gas (N_2_) flow rate of 80 arbitrary units (a.u.). The instrument was externally calibrated with appropriate standards.

Chromatographic separation of GAs was performed at ambient temperature on a Supelcosil LC-ABZ, amide-C_16_ (5 µm, 250  ×  4.6 mm), equipped with a guard column of the same material (Supelco Inc., Bellefonte, PA, USA), and a mobile phase consisting of 0.1% HCOOH in H_2_O (solvent A) and MeOH (solvent B). The following gradient at 0.8 mL/min was applied: 30–43% B in 0–8 min; 43–60% B in 8–20 min and 60% B in 20–24 min. Prior to the next injection, the column was equilibrated for 6 min. The injection volume was 20 μL and the flow to the source was reduced to 200 μL/min by a post-column splitter. Mass spectrometric data were acquired in positive ion mode while scanning *m/z* 50–1300 at a rate of 2 scans/s.

For the high-resolution MS/MS experiments by infrared multiphoton dissociation (IRMPD), precursor ions were isolated in the linear ion trap (LIT) and transferred into the ICR cell. The duration of laser irradiation was adapted to generate optimal fragmentation and varied between 100 and 350 ms. Typically, a medium IRMPD pulse, of 290 ms at 100% energy was applied as photon irradiation for GAs. Data were collected in full MS scan mode and processed post-acquisition to reconstruct the elution profile for the ions of interest with a given *m/z* value and tolerance. The simplest method to identify analytes by eXtracted Ion Chromatograms (XICs) in LC–FTICR-MS was used. The reduction of the interferences in the XICs significantly facilitates the identification of potential GAs, including the minor or uncommon compounds, which otherwise could have been missed. Data were acquired and processed by the Xcalibur software package (version 2.0 SR1; Thermo Fisher Scientific). The chromatographic raw data were imported, elaborated, and plotted using SigmaPlot 10.0 software (Systat Software, Inc., London, UK). Precursor and product ion structures were drawn by ChemDraw Pro 14.0 (CambridgeSoft Corporation, Cambridge, MA, USA). 

### 4.4. Acetylcholinesterase (AChE) Inhibitory Activity

The inhibition of AChE activity was determined based on Ellman’s method, as previously reported by [43]. For this assay, 25 μL of acetylthiocholine iodide (15 mM), 125 μL of DTNB (3 mM), 25 μL of buffer B (50mM Tris-HCl, pH 8 containing 0.1% BSA) and 50 μL of each test extract solution at the different concentrations were mixed. The mixture was incubated for 10 min at 37 °C. The reaction was started by adding 25 μL of 0.44 U/mL AChE. The absorbance was measured at 405 nm for 10 min and the rates of reactions were calculated by SpectroStar Nano. 

### 4.5. Antioxidant Activity

#### 4.5.1. Radical Scavenging Activity

The radical scavenging activity was measured using 2,2-diphenyl-1-picrylhydrazyl radical (DPPH) [13]. Several concentrations (from 5.0 mg mL^−1^ to 0.3 mg mL^−1^) of extracts (50 µL) were added to 200 µL of methanol DPPH solution (100 µM). Absorbance was read at 515 nm after 30 min of incubation in the darkness. Results were expressed as mg of Trolox equivalent (TE) per mg of dried extract.

#### 4.5.2. Inhibition of Lipid Peroxidation

The inhibition of lipid peroxidation was tested by beta carotene bleaching assay (BCB). For the analysis, 950 µL of beta carotene emulsion (0.4% *w/w*) was added to 50 µL of extract (0.25 mg mL^−1^). the beta carotene solution was prepared as previously reported [44]. BHT was used as a positive control and results were expressed as a percentage of antioxidant activity (% A.A.) [45].

### 4.6. Statistical Analysis 

The experimental results were expressed as mean ± standard deviation (SD) [46] of three independent replicates (n = 3). Data were analysed by Graph Pad version 5 and they were subjected to one-way analysis of variance (ANOVA) and differences between samples were determined by Tukey test, *p* values less than 0.05 were considered statistically significant.

## Figures and Tables

**Table 1:** Peak number, common name, molecular formulae, monoisotopic exact value and retention time of the glycoalkaloids detected in the two eggplant genotypes analysed.

Peak Number	Common Name	Molecular Formulae	Monoisotopic Exact Value [M+H]^+^ (*m/z*) (Δm) ^a^	Retention Time (Rt, min)	Genotype
1	Solanidenetriol chacotriose	C_45_H_71_NO_16_	882.48456 (−0.4)	5.6	Mir, Sen
2	Solanidenediol chacotriose	C_45_H_71_NO_15_	866.48965 (0.5)	6.0	Mir, Sen
3	Dehydrosolamargine	C_45_H_71_NO_15_	866.48965 (−0.2)	9.2	Mir, Sen
4	Solanandaine isomer I	C_45_H_73_NO_16_	884.50021 (0.4)	7.0	Mir, /
5	Solanandaine	C_45_H_73_NO_16_	884.50021 (−0.4)	8.2	Mir, Sen
6	Solasonine (spirosolenol solatriose)	C_45_H_73_NO_16_	884.50021 (−0.2)	13.6	Mir, Sen
7	Robenoside B (solanidenediol chacotriose)	C_45_H_73_NO_17_	900.49513 (0.6)	7.3	Mir, Sen
8	Malonyl- solanidenediol chacotriose	C_48_H_73_NO_18_	952.49004 (−0.5)	9.5	Mir, Sen
9	Solamargine Isomer (spirosolenol chacotriose)	C_45_H_73_NO_15_	868.50530 (1.2)	7.6	Mir, /
10	Solamargine (spirosolenol chacotriose)	C_45_H_73_NO_15_	868.50530 (1.3)	14.3	Mir, Sen
11	Solanidatetraenol chacotriose	C_45_H_67_NO_15_	862.45835 (0.3)	9.7	Mir, Sen
12	Malonyl-solanandaine	C_48_H_76_NO_19_	970.50061 (0.7)	11.9	Mir, Sen
13	Malonyl- solanidatetraenol chacotriose	C_48_H_69_NO_18_	948.45874 (−0.9)	13.4	Mir, Sen
14	Arudonine	C_50_H_82_NO_19_	1000.54756 (0.2)	14.1	Mir, Sen
15	Malonyl-solamargine isomer	C_48_H_76_NO_18_	954.50569 (1.2) 954.50569 (−0.5)	11.3	Mir, /
16	Malonyl-solamargine	C_48_H_76_NO_18_		18.5	Mir, Sen
17	Solanandaine Isomer II (spirosolendiol chacotriose)	C_45_H_73_NO_16_	884.50021 (−0.9)	9.5	Mir, Sen
18	Solanandaine Isomer III (spirosolendiol chacotriose)	C_45_H_73_NO_16_	884.50021 (−0.3)	10.1	Mir, Sen
19	Robenoside B Isomer (solanidenediol chacotriose)	C_45_H_73_NO_17_	900.49513 (1.3)	8.6	/Sen

^a^ Mass error in part per million (ppm) =10^6^ × (accurate mass-exact mass)/exact mass.

## Supplementary Materials

The following are available online at https://www.mdpi.com/2072-6651/11/4/230/s1, Figure S1: High-resolution IRMPD FTICR mass spectrum of the protonated Robenoside B at *m/z* 900, Figure S2: High-resolution IRMPD FTICR mass spectrum of the protonated arudonine at *m/z* 1000, Figure S3: High-resolution IRMPD FTICR mass spectra of the protonated solanandaine (A) at *m/z* 884 and malonyl-solanandaine (B) at *m/z* 970, Figure S4: Inhibition of Acetylcholinesterase (AChE) enzyme by Galantamine and Solamargine, Table S1: IRMPD FTICR MS product ions obtained from glycoalkaloids and malonyl glycoalkaloid and identified in S. melongena pulp by high-resolution LC-ESI-IRMPD-FTICR MS, Table S2: Fruit morphological traits of Mirabella and Bianca di Senise eggplants.
