# Supplementary material for: A Complete Survey of Glycoalkaloids Using LC-FTICR-MS and IRMPD in a Commercial Variety and a Local Landrace of Eggplant (Solanum melongena L.) and their Anticholinesterase and Antioxidant Activities
Source: Toxins (Basel). 2019 Apr 19;11(4):230. doi: 10.3390/toxins11040230 (PMC6521288; doi:10.3390/toxins11040230)
Supplement: Supplementary file 1 [file toxins-11-00230-s001.pdf]

Supplementary Materials: A complete survey of glycoalkaloids using LC-FTICR-MS and IRMPD in a commercial variety and a local landrace of eggplant (*Solanum melongena* L.) and their anticholinesterase and antioxidant activities

Filomena Lelario, Susanna De Maria, Anna Rita Rivelli, Daniela Russo, Luigi Milella, Sabino Aurelio Bufo and Laura Scrano

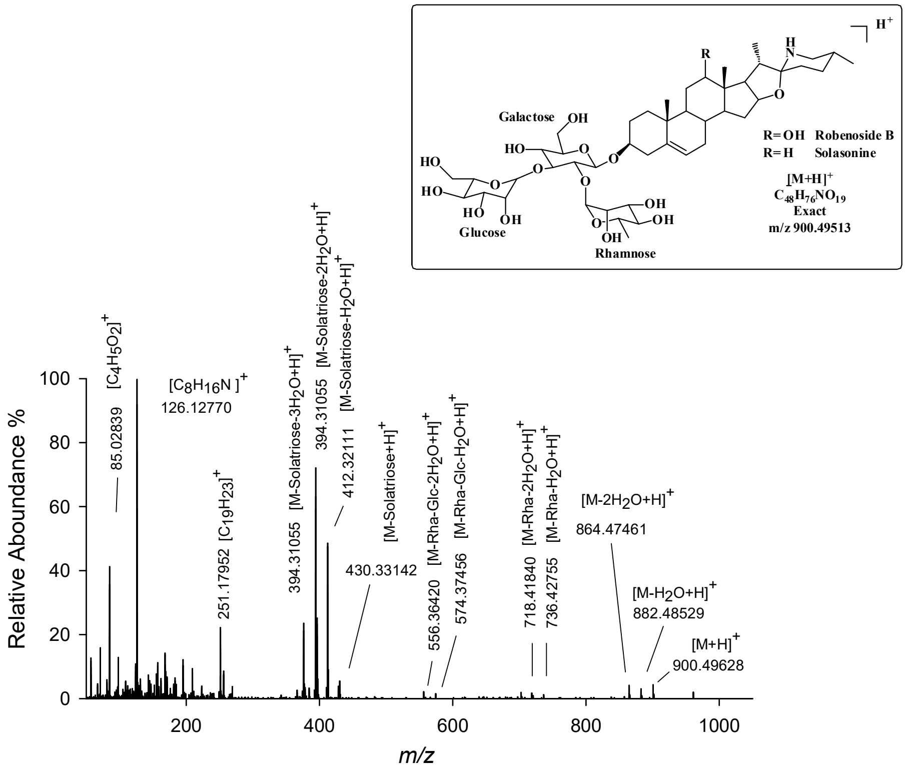

Figure S1. High-resolution IRMPD FTICR mass spectrum of the protonated Robenoside B at *m/z* 900. Following transfer to the ICR cell, precursor ion populations were photon-irradiated for 290 ms at 100% laser power.

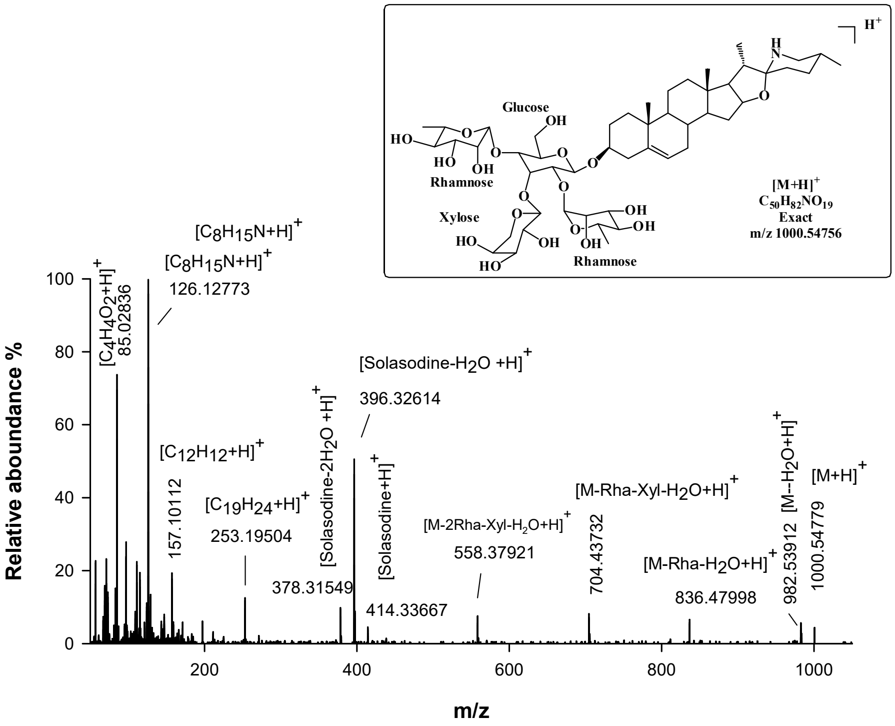

Figure S2. High-resolution IRMPD FTICR mass spectrum of the protonated arudonine at *m*/*z* 1000. Following transfer to the ICR cell, precursor ion populations were photon-irradiated for 290 ms at 100% laser power.

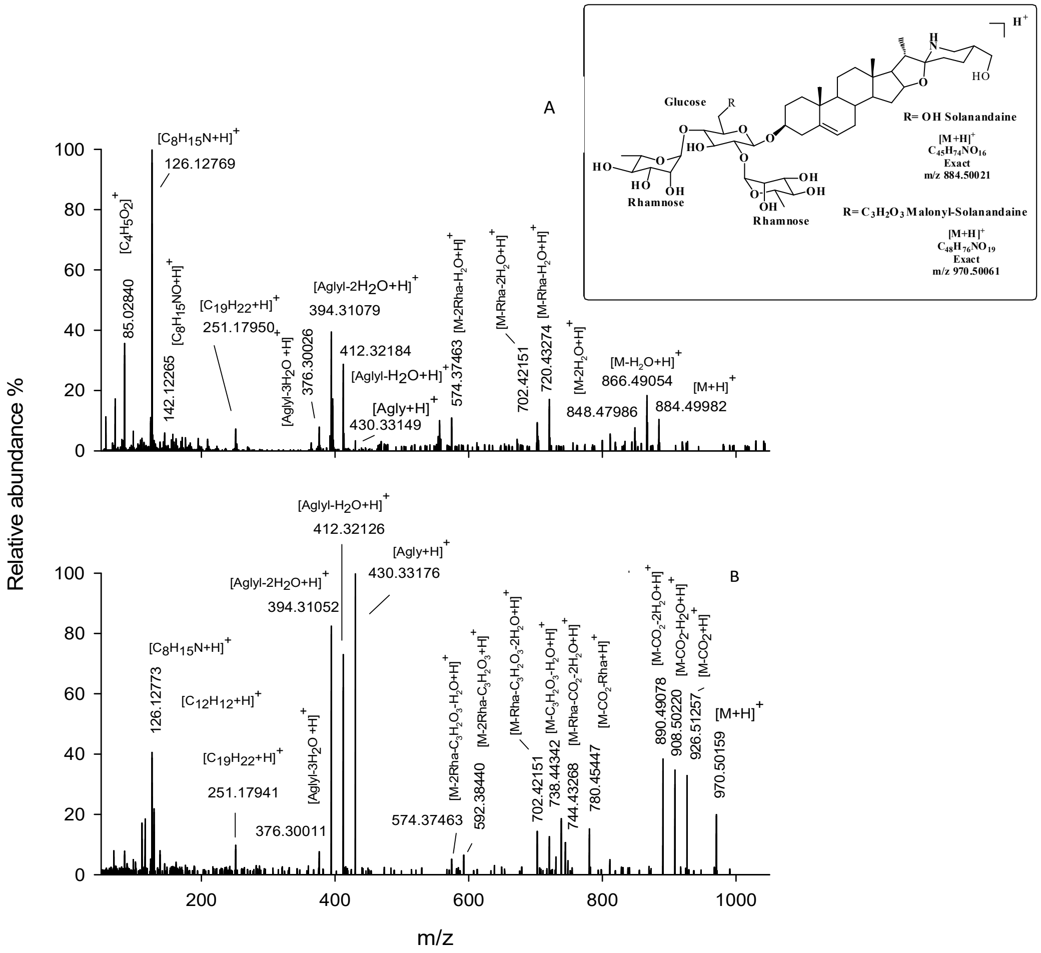

Figure S3. High-resolution IRMPD FTICR mass spectra of the protonated solanandaine (A) at *m*/*z* 884 and malonyl-solanandaine (B) at *m*/*z* 970. Following transfer to the ICR cell, precursor ion populations were photon-irradiated for 290 ms at 100% laser power.

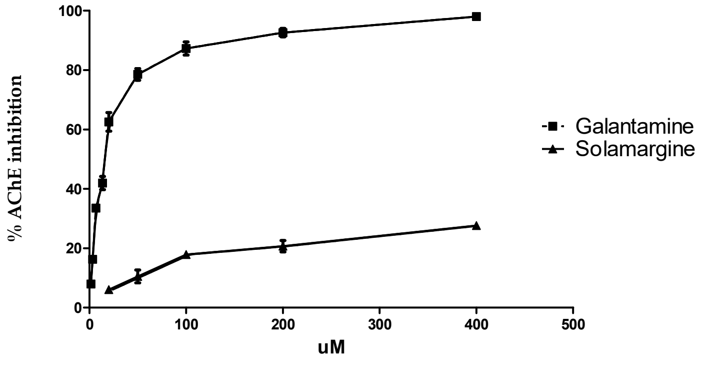

Figure S4. Inhibition of Acetylcholinesterase (AChE) enzyme by Galantamine and Solamargine.

Table S1. IRMPD FTICR MS product ions obtained from glycoalkaloids and malonyl glycoalkaloid and identified in *S. melongena* pulp by high resolution LC-ESI-IRMPD-FTICR MS.

Compound	Chemical formula as protonated molecule, [M+H]$^+$	Precursor Ion Accurate *m/z*	Main IRMPD MS/MS product ions (accurate *m/z*)[a] and mass error (ppm)[b]
1)	[$C_{45}H_{72}NO_{16}$]$^+$	882.48419	864.47543 ($C_{45}H_{70}NO_{15}^+$, 1.6), 718.41730($C_{39}H_{60}NO_{11}^+$, 0.7), 572.35901 ($C_{33}H_{50}NO_7^+$, 1.5), 428.31628 ($C_{27}H_{42}NO_3^+$, 0.8), 410.30557 ($C_{27}H_{40}NO_2^+$, 0.5), 392.29504 ($C_{27}H_{38}NO^+$, 0.6), 374.28439($C_{27}H_{36}N^+$, 0.4), 267.17441 ($C_{19}H_{23}O^+$, 0.2), 126.12770 ($C_8H_{16}N^+$, −0.2)
2)	[$C_{45}H_{72}NO_{15}$]$^+$	866.49005	720.43188 ($C_{39}H_{62}NO_{11}^+$, 0.2), 574.37402 ($C_{33}H_{52}NO_7^+$, 0.3), 412.31111 ($C_{27}H_{42}NO_2^+$, 0.2), 394.31052 ($C_{27}H_{40}NO^+$, 0.1), 182.15393 ($C_{11}H_{20}NO^+$, −0.05), 157.10114 ($C_{12}H_{13}^+$, −0.3), 85.02840 ($C_4H_5O_2^+$, −0.1)
3)	[$C_{45}H_{72}NO_{15}$]$^+$	866.48950	848.48059 ($C_{45}H_{70}NO_{14}^+$,1.5), 720.43060 ($C_{39}H_{62}NO_{11}^+$,−1.6), 702.42242($C_{39}H_{60}NO_{10}^+$−1.4), 574.37469 ($C_{33}H_{52}NO_7^+$, 1.1), 558.37952($C_{33}H_{50}NO_6^+$, 1.1), 556.36407($C_{33}H_{50}NO_6^+$, 1.4), 412.32129 ($C_{27}H_{42}NO_2^+$, 0.7), 396.32632 ($C_{27}H_{42}NO^+$, 0.6), 394.31067 ($C_{27}H_{40}NO^+$, 0.6), 376.30002 ($C_{27}H_{38}N^+$, −1.0)
4)	[$C_{45}H_{74}NO_{16}$]$^+$	884.50055	866.49023 ($C_{45}H_{72}NO_{15}^+$, 0.6), 412.32104 ($C_{27}H_{42}NO_2^+$, 0.08), 394.31076 ($C_{27}H_{40}NO^+$, 0.6), 271.20560 ($C_{19}H_{27}O^+$, −0.2), 253.19504 ($C_{19}H_{25}^+$, −0.15), 157.10112 ($C_{12}H_{13}^+$, −0.4), 85.02837 ($C_4H_5O_2^+$, −0.4)
5)	[$C_{45}H_{74}NO_{16}$]$^+$	884.49982	866.50575($C_{45}H_{72}NO_{15}^+$, 1.0), 720.43368($C_{39}H_{62}NO_{11}^+$,2.7), 430.33249 ($C_{27}H_{44}NO_3^+$, 2.1), 412.32184, 2.0), 394.311116($C_{27}H_{40}NO^+$, 1.8), 376.00063($C_{27}H_{38}N^+$, 1.9), 364.26401 ($C_{25}H_{34}NO^+$, 1.4), 251.17964 ($C_{19}H_{23}^+$, 0.8), 126.12769 ($C_8H_{16}N^+$, −0.3)
6)	[$C_{45}H_{74}NO_{16}$]$^+$	884.50000	866.50500 ($C_{45}H_{72}NO_{15}^+$, 0.1), 720.43324($C_{39}H_{62}NO_{11}$, 2.1), 558.37856 ($C_{33}H_{52}NO_6$, −0.6), 414. 33592($C_{27}H_{44}NO_2$, −1.7), 396.32658($C_{27}H_{42}NO$, 1.2), 253.19576($C_{19}H_{25}$, 2.7), 157.10123 ($C_{12}H_{13}$, 0.3), 126.12772($C_8H_{16}N$, 1.4), 85.02845($C_4H_5O_2$,0.5)
7)	[$C_{45}H_{74}NO_{17}$]$^+$	900.49607	882.48529 ($C_{45}H_{72}NO_{16}$, 0.8), 864.47461 ($C_{45}H_{70}NO_{15}$, 0.7), 736.42755(), 718.41640 ($C_{39}H_{60}NO_{11}$, 0.4), 574.37456 ($C_{33}H_{52}NO_6$, 1.3), 556.36420($C_{33}H_{50}NO_5$, 1.7), 430.33142 ($C_{27}H_{44}NO_3$, −0.8), 412.32111($C_{27}H_{42}NO_2$, 0.2), 394.31055 ($C_{27}H_{40}NO$, 0.3), 376.26370 ($C_{26}H_{34}NO$, 0.6), 251.17952 ($C_{19}H_{23}$, 0.4), 126.12770 ($C_8H_{16}N$, 0.04), 85.02839 ($C_4H_5O_2$, −0.02)
8)	[$C_{48}H_{74}NO_{18}$]$^+$	952.48956	908.50000($C_{47}H_{74}NO_{16}$, −0.2), 890.48959($C_{47}H_{72}NO_{15}$, −0.2), 762.44232 ($C_{41}H_{46}NO_{12}$, 0.02), 720.43188($C_{39}H_{62}NO_{11}$, 0.2), 574.37378($C_{33}H_{52}NO_7$, −0.08), 412.32077($C_{27}H_{42}NO_2$, −0.5), 394.31030 ($C_{27}H_{40}NO$, −0.4), 162.12762 ($C_{11}H_{16}N$, −0–6), 85.02835($C_4H_5O_2$, −0.6)
9)	[$C_{45}H_{74}NO_{15}$]$^+$	868.50641	850.49524 ($C_{45}H_{72}NO_{14}^+$, 0.6), 704.44912 ($C_{39}H_{62}NO_{10}^+$, 1.6), 414.33682 ($C_{27}H_{44}NO_2^+$, 0.4), 396.32608 ($C_{27}H_{42}NO^+$, −0.02), 378.31564 ($C_{27}H_{40}N^+$, 0.3), 253.19507 ($C_{19}H_{25}^+$, 0.03),
10)	[$C_{45}H_{74}NO_{15}$]$^+$	868.50573	850.50995 ($C_{45}H_{72}NO_{14}^+$, 1.6), 704.44910 ($C_{39}H_{62}NO_{10}^+$, 1.6), 558.37844 ($C_{33}H_{52}NO_6^+$, −0.8), 414.33688 ($C_{27}H_{44}NO_2^+$, 0.5), 396.32617 ($C_{27}H_{42}NO^+$, 0.2), 378.31558 ($C_{27}H_{40}N^+$, 0.1), 271.2056 ($C_{19}H_{27}O^+$, 0.2), 253.19508 ($C_{19}H_{25}^+$, 0.02), 197.13243 ($C_{15}H_{17}^+$, −0.2), 157.10110 ($C_{12}H_{13}^+$, −0.5), 147. 06511 ($C_6H_{11}O_4^+$, −0.5), 143.08546 ($C_{11}H_{11}^+$, −0.5), 126.12766 ($C_8H_{16}N^+$, −0.6), 85.02835 ($C_4H_5O_2^+$, −0.6)
11)	[$C_{45}H_{68}NO_{15}$]$^+$	862.45864	716.39897 ($C_{39}H_{58}NO_{11}^+$, −2.0), 570.34136 ($C_{33}H_{48}NO_7^+$, −2.0), 408.28930 ($C_{27}H_{38}NO_2^+$, −1.0), 390.27904($C_{27}H_{36}NO^+$, −0.3), 376.26392($C_{26}H_{34}NO^+$, 1.1)

12)	$[C_{48}H_{76}NO_{19}]^+$	970.50130	926.51257 ($C_{47}H_{76}NO_{17}^+$, −0.9), 908.50220 ($C_{47}H_{76}NO_{17}^+$, −0.9), 890.49078($C_{47}H_{72}NO_{15}^+$, 1.3), 780.45247($C_{41}H_{66}NO_{13}^+$, −0.5), 744.43268($C_{41}H_{62}NO_{11}^+$, 1.2), 702.42151($C_{39}H_{60}NO_{10}^+$, 0.4), 430.33179($C_{27}H_{44}NO_3^+$, 0.5), 412.32126($C_{27}H_{42}NO_2^+$, 0.6), 394.31052 ($C_{27}H_{40}NO^+$, 0.2), 376.30011($C_{26}H_{34}NO^+$,0.6), 251.17941 ($C_{19}H_{23}^+$, 0.05), 126.12772 ($C_8H_{16}N^+$, −0.02)
13)	$[C_{48}H_{70}NO_{18}]^+$	948.45788	904.46846 ($C_{47}H_{70}NO_{16}^+$, −0.5), 862. 45704 ($C_{45}H_{68}NO_{15}^+$, 1.5), 844.44654 ($C_{45}H_{66}NO_{14}^+$, −1.5), 758.44888 ($C_{41}H_{60}N0_{12}^+$, 2.0), 716.39987 ($C_{39}H_{58}NO_{11}^+$, −0.8), 408.28955 ($C_{27}H_{38}NO_2^+$, −0.4), 390.27852 ($C_{27}H_{36}NO^+$, 1.8), 376.26380 ($C_{26}H_{34}NO^+$, 0.8)
14)	$[C_{50}H_{82}NO_{19}]^+$	1000.54779	982.53912($C_{50}H_{80}NO_{18}^+$, 2.1), 836.47998($C_{44}H_{70}NO_{14}^+$, 1.0), 704.43732 ($C_{39}H_{62}NO_{10}^+$, 0.7), 558.37921 ($C_{33}H_{52}NO_{10}^+$, 0.5), 414.33667($C_{27}H_{44}NO_2^+$, 0.03), 396.32614($C_{27}H_{42}NO^+$, 0.1), 378.31549($C_{27}H_{40}N^+$, −0.1), 253.19504 ($C_{19}H_{25}^+$, −0.1), 157.10112 ($C_{12}H_{13}^+$, −0.3), 126.12766 ($C_8H_{16}N^+$, −0.5)
15)	$[C_{48}H_{76}NO_{18}]^+$	954.50584	910.51624 ($C_{47}H_{76}NO_{16}^+$, 0.4), 892.50586 ($C_{47}H_{74}NO_{15}^+$, 0.4), 764.45892 ($C_{41}H_{66}NO_{12}^+$, 0.1), 746.44781 ($C_{41}H_{64}NO_{11}^+$, 0.6), 722.44781 ($C_{39}H_{64}NO_{11}^+$, 0.6), 576.38971 ($C_{33}H_{54}NO_7^+$, 0.4), 558.37909 ($C_{33}H_{52}NO_6^+$, 0.3), 414.33670 ($C_{27}H_{44}NO_2^+$, 0.1), 396.32611 ($C_{27}H_{42}NO^+$, 0.04), 378.31555 ($C_{27}H_{40}NO^+$, 0.06), 271.20587($C_{19}H_{27}O^+$, 0.8)
16)	$[C_{48}H_{76}NO_{18}]^+$	954.50525	910.51751 ($C_{47}H_{76}NO_{16}^+$, 1.8), 892.50543($C_{47}H_{74}NO_{15}^+$, 0.1), 764.45806(0.1), 746.44784($C_{41}H_{66}NO_{12}^+$, 0.6), 722.44865 ($C_{39}H_{64}NO_{11}^+$, 1.7), 576.38941($C_{33}H_{54}NO_7^+$,−0.1), 558.37898($C_{33}H_{52}NO_6^+$, 0.1), 414.33672($C_{27}H_{44}NO_2^+$,0.1), 396.32690($C_{27}H_{42}NO^+$, 2.0), 378.31576 ($C_{27}H_{40}NO^+$,0.6), 253.19502 ($C_{19}H_2^+$, −0.2)
17)	$[C_{45}H_{74}NO_{16}]^+$	884.49939	866.48907 ($C_{45}H_{72}NO_{15}^+$, −0.7), 848.47858 ($C_{45}H_{70}NO_{14}^+$, −0.6), 720.43134 ($C_{39}H_{62}NO_{11}^+$, −0.6), 574.37366 ($C_{33}H_{52}NO_7^+$,−0.3), 430.33118 ($C_{27}H_{44}NO_3^+$, −0.9), 412.32077($C_{27}H_{42}NO_2^+$, −0.6), 394.31021($C_{27}H_{40}NO^+$, −0.6), 376.29959($C_{27}H_{38}N^+$, −0.7), 251.17924($C_{19}H_{23}^+$, −0.7), 168.13818($C_{10}H_{18}NO^+$, −0.6), 126.12765 ($C_8H_{16}N^+$, −0.6)
18)	$[C_{45}H_{74}NO_{16}]^+$	884.49994	866.48914 ($C_{45}H_{72}NO_{15}^+$, −0.6), 720.43140 ($C_{39}H_{62}NO_{11}^+$, −0.5), 430.33173($C_{27}H_{44}NO_3^+$, 0.4), 412.32098 ($C_{27}H_{42}NO_2^+$, −0.05), 394.31042($C_{27}H_{40}NO^+$, −0.04), 376.29977($C_{27}H_{38}N^+$, −0.3), 251.17937($C_{19}H_{23}^+$, −0.2), 162.12770($C_{11}H_{16}N^+$, −0.2), 126.12771($C_8H_{16}N^+$, −0.1)
19)	$[C_{45}H_{74}NO_{17}]^+$	900.49628	882.48529 ($C_{45}H_{72}NO_{16}^+$, 0.8), 864.47461 ($C_{45}H_{70}NO_{15}^+$, 0.7), 718.41699($C_{39}H_{60}NO_{11}^+$, 1.2), 430.33124 ($C_{27}H_{44}NO_3^+$, −0.8), 412.32111($C_{27}H_{42}NO_2^+$, 0.2), 394.31055 ($C_{27}H_{40}NO^+$, 0.3), 251.17952 ($C_{19}H_{23v}$, 0.4), 126.12773 ($C_8H_{16}N^+$, 0.04), 85.02840 ($C_4H_5O_2^+$, −0.02)

[a] Mass error in part per million (ppm) = 10^6 * (accurate mass-exact mass)/exact mass. [b] Significant signals with signal-to-noise ratio 5 or higher were taken into consideration.

Table S2. Fruit morphological traits of Mirabella and Bianca di Senise eggplants.

**Traits and units**	**Eggplant genotype**	
	**Mirabella**	**Bianca di Senise**
Color	dark purple	white with purple streaks
Length (cm)	11.1 ± 0.5	15.1 ± 0.4
Diameter at the middle (cm)	6.6 ± 0.5	8.4 ± 0.2
Fresh weight (g.)	152.6 ± 18	452.8 ± 54
Pulp dry matter (%)	11.5 ± 0.6	9.3 ± 0.3
Peel dry matter (%)	9.3 ± 0.1	7.0 ± 0.2
